# Validation of Gene Expression Patterns for Oral Feeding Readiness: Transcriptional Analysis of Set of Genes in Neonatal Salivary Samples

**DOI:** 10.3390/genes15070936

**Published:** 2024-07-18

**Authors:** Leonardo Henrique Ferreira Gomes, Andressa Brito Marques, Isabel Cristina de Meireles Dias, Sanny Cerqueira de O. Gabeira, Tamara Rosa Barcelos, Mariana de Oliveira Guimarães, Igor Ribeiro Ferreira, Letícia Cunha Guida, Sabrina Lopes Lucena, Adriana Duarte Rocha

**Affiliations:** 1 Instituto Nacional da Saúde da Mulher, da Criança e do Adolescente Fernandes Figueira–Fundação Oswaldo Cruz, Rio de Janeiro 22250-020, Brazil; leonardo.henrique@fiocruz.br (L.H.F.G.);; 2 Rural and Remote Support Services, Department of Health, Integrated Cardiovascular Clinical Network SA, Adelaide, SA 5042, Australia

**Keywords:** neonatal, salivary samples, gene expression, oral feeding

## Abstract

Background: Neonatal health assessment is crucial for detecting and intervening in various disorders. Traditional gene expression analysis methods often require invasive procedures during sample collection, which may not be feasible or ideal for preterm infants. In recent years, saliva has emerged as a promising noninvasive biofluid for assessing gene expression. Another trend that has been growing is the use of “*omics*” technologies such as transcriptomics in the analysis of gene expression. The costs for carrying out these analyses and the difficulty of analysis make the detection of candidate genes necessary. These genes act as biomarkers for the maturation stages of the oral feeding issue. Methodology: Salivary samples (n = 225) were prospectively collected from 45 preterm (<34 gestational age) infants from five predefined feeding stages and submitted to RT-qPCR. A better description of the targeted genes and results from RT-qPCR analyses were included. The six genes previously identified as predictive of feeding success were tested. The genes are *AMPK*, *FOXP2*, *WNT3*, *NPHP4*, *NPY2R*, and *PLXNA1*, along with two reference genes: *GAPDH* and *18S*. RT-qPCR amplification enabled the analysis of the gene expression of *AMPK*, *FOXP2*, *WNT3*, *NPHP4*, *NPY2R*, and *PLXNA1* in neonatal saliva. Expression results were correlated with the feeding status during sample collection. Conclusions: In summary, the genes *AMPK*, *FOXP2*, *WNT3*, *NPHP4*, *NPY2R*, and *PLXNA1* play critical roles in regulating oral feeding and the development of premature infants. Understanding the influence of these genes can provide valuable insights for improving nutritional care and support the development of these vulnerable babies. Evidence suggests that saliva-based gene expression analysis in newborns holds great promise for early detection and monitoring of disease and understanding developmental processes. More research and standardization of protocols are needed to fully explore the potential of saliva as a noninvasive biomarker in neonatal care.

## 1. Introduction

Preterm infants often have a history of prolonged hospitalization and invasive procedures. Concurrently, they must acquire skills for successful oral feeding before discharge. It is not a simple task, as the success of oral feeding involves the maturation and integration of the entire nervous, sensory, muscular, and digestive systems and is not without risks [1,2]. According to Griffith et al. (2019) [2], the term “successful oral feeding” is frequently used in clinical practice and research. However, this term needs to be more consistently defined since it affects the ability to adequately assess the success of oral feeding, identify risk factors, and implement interventions in clinical practice and research.

Unsuccessful oral feeding can result in a range of morbidities, including choking, aspiration, bradycardia, desaturation, food aversions, and short- and long-term neurological complications [3]. Newborns rely on the subjective interpretation of healthcare professionals to determine when it is safe to eat orally, often based on physiological signs such as oxygen saturation, coughing, and choking [4]. A new reality in Brazilian neonatal intensive care units (NICUs) is the introduction of oral feeding for preterm newborns based on gestational age (GA) and weight criteria.

There is no universal protocol for suction assessment, but some authors have proposed guidelines and protocols [4,5,6,7,8,9,10]. There is a significant need to develop new noninvasive neonatal monitoring techniques to identify new biomarkers that help elucidate biological functions and predict disease states.

In this regard, Maron et al. [11] identified some genes more predictive of feeding success, representing a diverse range of biological systems, including sensory integration (Nephrocystin 4—*NPHP4*, Plexin A1—*PLXNA1*), hypothalamic regulation of feeding (neuropeptide Y receptor Y2—*NPY2R*), facial development (proto-oncogene—*WNT3*) and energy expenditure (5′ adenosine monophosphate-activated protein kinase—*AMPK*). The Forkhead Box P2 (*FOXP2*) gene is known for its crucial role in the development of language and speech, but recent studies have suggested that it may be involved in other developmental processes, including feeding maturity. A mature oral feeding pattern was predicted when three genes showed negative gene expression (*NPHP4*, *NPY2R*, and *WNT3*), and two genes showed positive gene expression (*AMPK* and *PLXNA1*). These biomarkers were noninvasively monitored through less than 100 µL of saliva in newborns. The author concludes that this is the way forward for developing a diagnostic test to accurately and reliably predict oral readiness for feeding in this vulnerable population, thus significantly improving clinical care and reducing costs associated with feeding morbidities in preterm newborns [11].

A biomarker is an objectively measured and evaluated indicator of normal biological processes, pathogenic processes, or pharmacologic responses to therapeutic intervention [12]. In summary, biomarkers are entities within the body capable of providing impartial information regarding the current physiological state of a living organism [13]. Saliva is a potential source of biomarkers for diagnostic purposes. One of the main advantages of saliva is its noninvasive, easy sample collection, which can be performed even by untrained personnel. Additionally, sampling is quick and easy, making it advantageous for large-scale screenings of children, the elderly, or in cases where repeated samples are required [14].

Similar to blood, saliva is a complex fluid containing a variety of enzymes, hormones, antibodies, antimicrobial constituents, and growth factors [15]. Saliva and oral swabs have been employed in the detection of genetic material (DNA and RNA) and pathogen-specific antibodies in a range of viral infections, including dengue, hepatitis B, measles, rubella, and parvovirus B19 [16,17,18,19].

Salivary transcriptomic studies are a relatively new territory in salivary diagnostics. In these studies, the upregulation or downregulation of various gene transcripts may serve as specific biomarker indicators of particular diseases or conditions.

This analysis and diagnostic tool gains even more power as it does not require blood collection. Incorporating molecular techniques into conventional clinical approaches aims to improve the understanding of the complexity of oral feeding and contributes to making it as safe as possible.

Although saliva’s diagnostic potential is already known, the application of molecular saliva analyses in newborns has been slow to emerge. A limiting factor is the limited volume due to the scarcity of data. Our research focused on neonatal salivary gene expression of genes representing a wide range of biological functions necessary for the success of oral feeding.

## 2. Materials and Methods

### 2.1. Salivary Collection

Saliva samples from newborns (n = 225) were prospectively collected from 45 preterm (<34 gestational age) from five predefined feeding stages from newborns were collected at the following times: (1) Zero diet; (2) Partial enteral feeding; (3) Full enteral feeding; (4) Partial oral feeding; (5) Full oral feeding. Salivary samples were collected and processed using previously described techniques (11). Briefly, saliva was collected with a 1 mL syringe attached to a low-wall suction. Our hospital adheres to the principle of minimal handling of hospitalized children. All procedures are performed together. In this case, saliva collections were conducted during other procedures (such as diaper changes). During these times, it is common for the children to cry and produce more saliva. The preterm infant’s oropharynx was gently aspirated, and saliva was immediately stabilized in 500 µL of RNA protect Saliva (QIAGEN). This stabilizing agent disrupts gene expression changes, inhibits microbial overgrowth, and destroys ubiquitous RNases. Two saliva samples were collected from a single time point. The samples were stored and kept at −80 °C until the time of processing.

The study was conducted by the Declaration of Helsinki and approved by the Fernandes Figueira Institute IRB (CAAE: 45767015.0.0000.5269). The Informed Consent Statement was obtained from all guardians of subjects involved in the study. Written informed consent was obtained from the participants’ guardians to publish this document.

### 2.2. RNA Extraction

All procedures were performed in an RNase-free environment. RNA extraction from preterm saliva samples was carried out using the modified TRIzol (Thermo Fisher Scientific, Waltham, MA, USA) protocol described by Ghandi et al. (2020) [20].

Frozen saliva samples were thawed at room temperature. No procedure was used to expedite thawing to avoid RNA degradation. Approximately 1 mL samples were aliquoted into sterile, DNase- and RNase-free 1.7 mL Eppendorf tubes 3810X (Eppendor f Hamburg, Germany) and centrifuged at 16,100 RCF and 4 °C for 20 min. The salivary supernatant was not used for RNA preparation.

One milliliter of TRIzol reagent (Thermo Fisher Scientific, Waltham, MA, USA) was added to each pellet and pipetted several times, followed by vortexing for 20 s to homogenize. The samples were incubated at room temperature for 5 min. Then, 200 μL of chloroform (Sigma-Aldrich, St. Louis, MO, USA) was added to each tube and vortexed for 20 s, followed by incubation at room temperature for 5 min. The samples were centrifuged at 16,100 RCF for 20 min at 4 °C. Approximately 700 μL of the upper aqueous layer from each sample was carefully transferred into a new DNase- and RNase-free 1.7 mL Eppendorf tubes 3810X (Eppendorf, Hamburg, Germany). These chloroform steps were repeated twice, transferring smaller amounts of the upper aqueous layer each time: approximately 600 μL in the first repetition and 450–500 μL in the second. At this stage, 500 μL of cold isopropyl alcohol (Sigma-Aldrich) was added to each tube and vortexed for about 10 s. The tubes were incubated at −20 °C for at least 1 h to ensure RNA precipitation. Following incubation, the samples were centrifuged for 20 min at 4 °C at 16,100 RCF. The supernatant was removed and discarded. The pellet was washed with 1 mL of cold 80% molecular-grade ethanol and centrifuged at 16,100 RCF for 5 min at 4 °C. This step was repeated once more. The samples were briefly centrifuged, and excess ethanol was removed using a pipette. The pellet was air-dried at room temperature for at least 5 min, resuspended in 20 μL of DNase- and RNase-free water, and incubated in a 55°C water bath for 5 min. Finally, we briefly vortexed the samples and performed a quick spin to collect the sample at the bottom of the tube. RNA samples were stored at −80 °C.

The samples were analyzed for the expression of genes *NPY2R*, *AMPK*, Forkhead Box Protein P2 (*FOXP2*), *WNT3*, *NPHP4*, and *PLXNA1* as genes of interest and glyceraldehyde-3-phosphate dehydrogenase (*GAPDH*) and ribosomal 18S (*18S*) as housekeeping genes.

### 2.3. RNA Quantification

RNA concentration (ng/μL) was determined using a Qubit 2.0^TM^ Fluorometer with RNA Quantification, Broad Range Assay Kit^TM^ for Qubit, and a NanoDrop™ 2000 Spectrophotometer (Thermo Fisher Scientific, Wilmington, NC, USA). The RNA purity (260/280 and 260/230 ratios) was assessed by NanoDrop 2000 Spectrophotometer (Thermo Fisher Scientific, Wilmington, NC, USA).

### 2.4. Quantitative Reverse Transcription PCR (RT-qPCR)

The gene expression of *AMPK*, *FOXP2*, *WNT3*, *NPHP4*, *NPY2R*, *PLXNA1*, *18S*, and *GAPDH* genes was analyzed by quantitive reverse transcription PCR (RT-qPCR) for each collection stage. The primers used are described in the Supplementary Materials File S1.

The reverse transcription was carried out using the SuperScript III First-Strand Synthesis System for RT-qPCR (Invitrogen, Waltham, MA, USA), with an input of 500 ng of RNA and using a mix of 2 pmol of each gene-specific primer for cDNA synthesis. The steps were carried out following the manufacturer’s protocol.

The RT-qPCR was performed using a 7500 fast Real-Time PCR (Applied Biosystems, Foster City, CA, USA) using the Power SYBR Green PCR Master Mix (Applied Biosystems, Foster City, CA, USA) according to the manufacturer’s specifications. The reaction mixture was prepared containing 1 μL of cDNA from each sample mixed with 12.5 μL of 2× Power SYBR Green PCR Master Mix and 1 μL of each primer (200 nmolar/rx) in a final volume of 25 μL. The cDNA was amplified at 95 °C for 10 min followed by 40 cycles of 95 °C for 15 s and 60 °C for 1 min. cDNA dilution curves checked PCR efficiency and correlation coefficient. The efficiency exceeded 92%, and the correlation coefficient was ≥0.99 in all assays. A reverse transcription negative control (without reverse transcriptase) and a non-template negative control were included for each primer set to confirm genomic DNA’s absence and test for primer dimers and contamination in the reactions, respectively. A melting curve analysis ensured that only a single product was amplified. Relative mRNA levels were normalized against the GAPDH and 18S mRNA (internal reference). The 2^−ΔΔCt^ method was used for relative gene expression quantification in all samples [21,22]. The feeding stage 1 (zero diet) was a control situation. The geometric mean of the Ct values of both reference genes was used to calculate ΔCts with the following equation: ΔCt = (Mean Ct gene of interest − $\sqrt{\left(Ct\;GAPDH\times Ct\;18S\right)}$ [23]. All data were expressed as the mean ± SD of each stage of the samples from the children (each in triplicate).

### 2.5. Statistical Analysis

Differences were considered statistically significant when *p* < 0.001 (**), as determined by GraphPadPrism 5.0 software (Graph-Pad Software Inc., San Diego, CA, USA). Analyses were performed using one-way ANOVA test with Greenhouse–Geisser correction.

## 3. Results and Discussion

The average RNA concentration in Trizol samples, quantified by NanoDrop, was approximately 265.23 ng/μL (±40.91), compared to 111 ng/μL (±16.86) by Qubit (Supplementary Materials File S2). The A260/A280 absorbance ratio was 1.95 (0.04), whereas the A260/A230 ratio was 1.75 (±0.09).

All efforts were made to adhere to the Minimum Information for Publication of Quantitative Real-Time PCR Experiments (MIQE) guidelines [21,22]. Singleplex amplification by RT-qPCR was performed on all total RNA samples extracted for the genes *AMPK*, *FOXP2*, *WNT3*, *NPHP4*, *NPY2R*, and *PLXNA1*, along with two reference genes, *GAPDH* and *18S*. The reference genes were selected based on literature [24] reporting that each of these genes maintains a relatively constant expression level and consistent expression range at different postnatal ages. The reference gene *GAPDH* had expression levels in our samples (average Ct: 28.34), while 18S (average Ct: 26.21) (Supplementary Materials File S3). All 245 samples analyzed showed amplification with both reference genes. There was no amplification of the gene targets in the negative control wells. All primers used showed similar efficiency and only one peak in the melt curve, indicating amplification of a single product (Supplementary Materials File S4).

**Figure 1:**
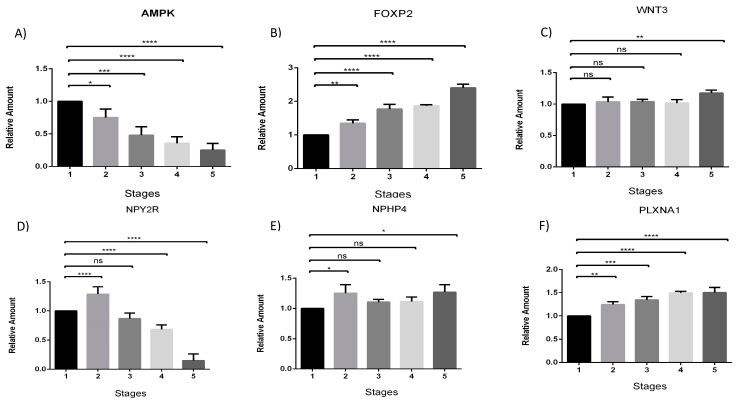
Analysis of gene expression of *AMPK*, *FOXP2*, *WNT3*, *NPY2R*, *NHPH4*, and *PLXNA1* at different feeding stages (**A**–**F** respectively). The data indicate the fold change in relative expression at different stages compared to the control condition (stage 1—zero diet), using the qRT-PCR technique. The numbers 1–5 represent the five predefined feeding stages from which the newborn samples were collected: (1) Zero diet; (2) Partial enteral feeding; (3) Full enteral feeding; (4) Partial oral feeding; (5) Full oral feeding. Analyses were performed using one-way ANOVA test. Error bars represent the standard deviation. Asterisks indicate the level of significance (* *p* < 0.05; ** *p* < 0.01; *** *p* < 0.001; **** *p* < 0.0001).

The expression profiles of the studied genes at different feeding stages were determined by the ΔΔCt method, and the results were expressed as fold change. The gene expression of *AMPK* decreased across feeding stages (Figure 1A). In contrast, the *FOXP2* gene exhibited an increasing pattern of gene expression, starting from stage 3 (full enteral feeding) and reaching approximately 3-fold in stage 5 (full oral feeding) compared to the control (zero diet) (Figure 1B). The gene expression profile of *WNT3* showed differences only in the last stage compared to the control situation (Figure 1C). The *NPHP4* gene exhibited both increase and decrease in expression elevation, with a significant increase in stages 2 (partial enteral feeding) and 5 (complete oral feeding) (Figure 1D). The *NPY2R* gene showed decreased expression patterns in stages 4 (partial oral feeding) and 5 (full oral feeding) (Figure 1D). Meanwhile, the *PLXNA1* gene showed increased gene expression from stage 2 (partial enteral feeding) (Figure 1E). The calculations of the test are presented in the Supplementary Material (Supplementary Materials File S5) with a different arrangement of gene expression.

Obtaining sufficient high-quality RNA is the initial step in conducting gene expression studies, particularly crucial when subsequent steps might compromise RNA quality—such as reverse transcription, qPCR, transcriptome analysis, and RNA-seq. While peripheral blood is commonly used, challenges arise in collecting samples from preterm infants and children and shipping materials from patients and family members residing in remote and inaccessible locations [25,26,27].

In this study, we focused on the gene expression of genes associated with a wide range of biological functions necessary for the success of oral feeding. Our results demonstrate that saliva-based gene expression analysis has a positive predictive value in determining the stages of feeding readiness in premature infants.

Determining feeding readiness in premature and newborn babies is a critical issue in neonatology as it is directly linked to the development and health of these vulnerable infants. The ability to accurately assess when a baby is ready to initiate oral feeding can significantly impact their nutritional progression and the risk of complications associated with premature feeding. In this context, gene expression has emerged as a promising tool for identifying biomarkers indicating feeding readiness in premature and newborn babies [28,29]. Omics approaches still need to be improved due to their high cost and difficulty in processing and interpreting the data [30].

Maheshwari et al. (2017) [31] investigated gene expression profiles in buccal epithelial cells of premature newborns during the transition from enteral to oral feeding. The researchers identified a specific genetic signature associated with oral neuromuscular maturation, indicating that the expression of specific genes may serve as predictive biomarkers of feeding readiness in premature babies. Additionally, some studies have explored the use of gene expression biomarkers in peripheral blood to assess feeding readiness in premature babies. Chawla et al. (2017) [32] investigated gene expression profiles in the blood cells of premature babies during the transition from enteral to oral feeding. The results suggested that specific genes related to gastrointestinal function and immune response may be helpful indicators of feeding readiness in premature babies.

Using a gene expression panel associated with critical roles in regulating oral feeding and the development of premature infants aims to identify a combination that provides assessable and measurable characteristics indicative of biological processes [23]. The gene expression panel should possess high specificity for the desired effect, reflecting the effect from the outset, being determinable, analyzable by noninvasive techniques, and exhibiting high sensitivity in the chosen biological fluid [32]. Thus, the more sensitive the biomarker, the earlier any metabolic alterations can be detected, allowing for intervention in the progression of effects and potentially preventing the emergence of an unfavorable clinical condition [30]. However, it is challenging for a single biomarker to possess all these characteristics; hence, the use of a gene panel.

Standardizing a set of genes that reflect a broad range of biological functions aims to develop an objective readiness analysis panel and reduce costs. The *NPY2R* gene encodes the neuropeptide Y receptor Y2 (NPY2R), a member of the G-protein coupled receptor family primarily involved in regulating energy homeostasis, food intake, and various neuroendocrine functions. It binds to neuropeptide Y (NPY) and peptide YY (PYY), crucial regulators of appetite and food intake. NPY2R plays a critical role in the central nervous system’s regulation of appetite, inhibiting NPY release upon activation in the hypothalamus to reduce appetite and food intake, contributing to energy balance. Additionally, NPY2R modulates energy expenditure, maintaining body weight stability. It also regulates various neuroendocrine pathways, influencing hormone release from the hypothalamus and pituitary gland, affecting functions like stress response, reproduction, and growth. Furthermore, NPY2R has been implicated in modulating anxiety and stress responses, exhibiting anxiolytic effects upon activation in specific brain regions. Studies suggest that variations in the *NPY2R* gene may affect feeding behavior by influencing neural responses to feeding and satiety sensation, indirectly impacting feeding patterns and nutritional development in premature infants [33,34,35,36,37].

The *AMPK* (AMP-activated protein kinase) gene is a central regulator of cellular metabolism, playing a crucial role in maintaining energy homeostasis at both cellular and systemic levels. It responds to changes in cellular energy levels by promoting processes that restore energy balance. *AMPK* is activated in response to an increased AMP/ATP ratio, signaling low cellular energy levels. Once activated, it promotes ATP production by stimulating fatty acid oxidation, glucose uptake, and glycolysis while inhibiting energy-consuming processes such as fatty acid synthesis, protein synthesis, and glycogen synthesis. AMPK also enhances insulin sensitivity and glucose uptake, particularly in skeletal muscle, and suppresses hepatic glucose production, helping to regulate blood glucose levels. Additionally, AMPK promotes mitochondrial biogenesis, increasing the cell’s oxidative capacity and improving energy efficiency. It also activates autophagy, a cellular recycling process critical for removing damaged components and ensuring cell survival under stress conditions. Activation of AMPK has been associated with anti-aging effects and extended longevity in various animal models. In premature infants, AMPK activation may be crucial to optimize nutrient utilization during oral feeding, ensuring an adequate energy supply for growth and development. The *AMPK* gene is a crucial regulator of cellular energy metabolism. In premature infants, AMPK activation may be crucial to optimize the utilization of nutrients during oral feeding, ensuring an adequate energy supply for growth and development [38,39,40,41,42].

The *FOXP2* gene encodes a transcription factor that plays a crucial role in the development and function of several tissues, including the brain. It is particularly well-known for its involvement in language and speech development. Mutations in the *FOXP2* gene have been linked to speech, language disorders, and orofacial motor function, highlighting its importance in these processes. FOXP2 influences the development of neural circuits that are essential for proper speech and language function. It is essential for the proper development of neural circuits in the brain, especially those related to language and speech, and regulates the expression of other genes involved in neural plasticity, synaptic function, and connectivity. Studies have shown that individuals with mutations in the *FOXP2* gene often exhibit speech and language deficits, such as developmental verbal dyspraxia, which affects the ability to coordinate the movements required for speech. Additionally, *FOXP2* is involved in the fine-tuning of motor skills necessary for articulate speech and is implicated in broader cognitive processes, including learning and memory, affecting the plasticity of neural networks fundamental for cognitive development [43,44,45,46,47].

The *WNT3* gene encodes a protein that is a member of the WNT family, a group of signal transduction pathways crucial for regulating embryonic development, cell growth, migration, and differentiation. WNT3 plays a vital role in the canonical Wnt/β-catenin signaling pathway, influencing various developmental processes and maintaining adult tissue homeostasis. It is essential for early embryonic development, particularly in forming the primitive streak, which establishes the body axis and germ layers in the developing embryo. WNT3 also regulates cell proliferation and differentiation during embryogenesis and in adult tissues, influencing the fate of various cell types and contributing to organ and tissue development. In neural development, WNT3 influences the patterning and growth of the nervous system, aiding in the differentiation of neural progenitor cells and the formation of neural circuits. In adult tissues, WNT3 signaling is crucial for maintaining and regulating stem cells, including hematopoietic and mesenchymal stem cells, sustaining the stem cell population, and regulating their differentiation. WNT3 is also involved in bone formation and homeostasis by regulating the differentiation of osteoblasts, which are responsible for bone synthesis, thus playing a role in bone growth and repair. In premature infants, altered expression of the *WNT3* gene may affect the formation and function of the gastrointestinal tract, impacting the ability for oral feeding and subsequent development. Understanding the role of WNT3 in these processes is essential, as it can provide insights into managing and improving outcomes for premature infants with feeding difficulties [48,49,50,51].

The *NPHP4* gene encodes the nephrocystin-4 protein, which plays a crucial role in the functioning and structural integrity of cilia, cellular organelles involved in signaling pathways, and cellular movement. Mutations in the *NPHP4* gene are associated with nephronophthisis, a genetic disorder that affects kidney function and can lead to end-stage renal disease. NPHP4 is a critical component of the ciliary protein complex, interacting with other nephrocystins to maintain the structural integrity and function of primary cilia, essential for cellular signaling and tissue homeostasis. It is crucial for kidney development and function, regulating the structure of renal tubules and the filtration process. Disruptions in NPHP4 can lead to nephronophthisis, characterized by fibrosis and cyst formation in the kidneys. Additionally, NPHP4 is involved in multiple cell signaling pathways, including Wnt and Hedgehog signaling, influencing cell proliferation and differentiation. NPHP4 interacts with other nephrocystins and proteins involved in ciliary function, such as NPHP1 and NPHP3, crucial for forming the ciliary transition zone, a barrier and signaling hub. Besides nephronophthisis, mutations in *NPHP4* are implicated in other ciliopathies, such as Bardet-Biedl syndrome and Senior-Loken syndrome, which exhibit overlapping phenotypes, including retinal degeneration, obesity, and polydactyly. In premature infants, altered expression of *NPHP4* may significantly impact renal function, potentially influencing the delicate balance of fluid and electrolytes essential for their growth and development. Disruptions in the *NPHP4* gene in these infants could lead to difficulties in maintaining proper kidney function, which is vital for effective nutrient absorption and waste elimination during critical periods of feeding and growth. Studies suggest that genetic variants in *NPHP4* may influence renal function in premature infants, indirectly affecting fluid and electrolyte balance during feeding and development. Understanding the role of *NPHP4* in these processes is essential for managing and improving outcomes for premature infants with renal and feeding difficulties [52,53,54,55,56].

The *PLXNA1* gene encodes plexin-A1, a protein that is part of the plexin family of receptors, primarily known for their roles in the nervous system. Plexin-A1 interacts with semaphorins, a group of proteins that guide axon growth and cell migration. The functions of PLXNA1 extend beyond neural development and include roles in the immune system and various cellular processes. PLXNA1, through its interaction with semaphorins, plays a crucial role in axon guidance essential for the proper wiring of the nervous system by directing axons to their appropriate targets, thus ensuring the correct formation of neural circuits. In premature infants, genetic variations in *PLXNA1* may affect the formation of neural connections necessary for effective sucking and swallowing during oral feeding. Additionally, PLXNA1 regulates cell migration and cytoskeletal dynamics, mediating signal transduction pathways that lead to changes in the actin cytoskeleton, which is crucial for cell movement and stability. In the immune system, *PLXNA1* influences the migration and activation of dendritic cells and T cells, modulating immune responses. It is also implicated in bone homeostasis through its role in osteoclast differentiation and function, regulating bone density and integrity. Aberrant expression of *PLXNA1* has been linked to various cancers, affecting cell migration and invasion, indicating its potential as a target for cancer therapy. Understanding the role of *PLXNA1* in these processes, especially in the context of premature infants, is essential for managing and improving outcomes for those with developmental challenges, highlighting its significance in neural development, immune function, and feeding capabilities [57,58].

The strength of this approach for assessing development in preterm infants lies not only in the fact that these genes are detectable in saliva and change with advanced feeding status. In reality, they provide a window into developmental areas that may be causing feeding delays. Additionally, the pleiotropy of these genes, which is the ability of a single gene to influence multiple phenotypic traits, adds a layer of complexity and relevance to the analysis.

For example, the statistically most significant predictor of oral feeding competence in this study is the decreased expression of the *NPY2R* gene, which regulates hunger signaling. Increased expression of *NPY2R* leads to satiety, while decreased expression results in hunger. The results of this study validate others (10, 14, 15) that demonstrated that the success of oral feeding can be predicted by the decreased expression of *NPY2R* in saliva, which likely signals a more mature gut-brain axis. Understanding that this could be the developmental milestone hindering feeding success can guide care practices.

On the other hand, the *PLXNA1* gene is involved in sensory integration and neurodevelopment. Infants with a more immature *PLXNA1* profile may benefit from more oral stimulation or the kangaroo method. Understanding these differences helps tailor care approaches and possibly reduce the time needed for oral feeding based on each infant’s developmental stage. Considering the pleiotropy of these genes further strengthens the understanding of the multiple roles they play in infant development.

Salivary transcriptomic studies have great potential, but the costs associated with “omics” and the complexity of the analyses are still prohibitive. The association of a group of genes can be useful in determining feeding stages with an analysis of a group of genes by RT-qPCR versus transcriptomic or RNAseq.

In summary, the genes *NPY2R*, *AMPK*, *FOXP2*, *WNT3*, *NPHP4*, and *PLXNA1* play critical roles in regulating oral feeding and the development of premature infants. Understanding the influence of these genes can provide valuable insights to improve nutritional care and support the development of these vulnerable babies.

## 4. Conclusions

Relying solely on clinical examination, determining feeding stages is challenging to diagnose in preterm infants. This fact can influence hospitalization duration and increase invasive procedures. The success of oral feeding encompasses the maturation and integration of the sensory, muscular, and digestive systems. Using a set of genes associated with these areas can act as biomarkers of feeding readiness in premature and newborn babies. Identifying molecular biomarkers can assist healthcare professionals in making more informed clinical decisions, enabling a personalized approach for feed and nutritional care for these fragile infants and bringing us closer to the concept of “successful oral feeding”.

## Data Availability

The datasets generated and analyzed during the current study are not publicly available due to patient data’s confidentiality and ethical aspects but are available from the corresponding author at a reasonable request.

## Supplementary Materials

The following supporting information can be downloaded at https://www.mdpi.com/article/10.3390/genes15070936/s1, File S1: Sequence of primers used in the study; File S2: RNA concentration data, 260/280 and 260/280absorbance ratios; File S3: Ct files; File S4: Melt curves of the PCR amplicons used in the RT-qPCR experiments. File S5: One Way ANOVA test calculations.

## Abbreviations

18S	ribosomal 18S gene
AMPK	5′ adenosine monophosphate-activated protein kinase gene
cDNA	complementary DNA
FOXP2	Forkhead Box Protein P2 gene
GA	gestational age neonatal intensive care units (NICUs)
GAPDH	glyceraldehyde-3-phosphate dehydrogenase gene
mRNA	messenger RNA
NICUs	neonatal intensive care units
NPHP4	Nephrocystin 4 gene
NPY2R	neuropeptide Y receptor 2 gene
PLXNA1	Plexin A1 gene
qPCR	quantitative polymerase chain reaction
RNA-seq	RNA sequencing
RT-qPCR	reverse transcription quantitative PCR
WNT3	proto-oncogene—WNT3
