# Supplementary material for: Validation of Gene Expression Patterns for Oral Feeding Readiness: Transcriptional Analysis of Set of Genes in Neonatal Salivary Samples
Source: Genes (Basel). 2024 Jul 18;15(7):936. doi: 10.3390/genes15070936 (PMC11275400; doi:10.3390/genes15070936)
Supplement: Supplementary file 1 [file genes-15-00936-s001.zip › File S1.pdf]

Additional File S1

Sequence of primers used in the study

Gene	Primer (5'-3')		Accession No
NPY	Foward Sequence	ACTCCTAGAGGTGAACTGGTCC	NM_000910
	Reverse Sequence	CATGGATCACCAAGGAGTTGCC	
AMPK	Foward Sequence	AGGAAGAATCCTGTGACAAGCAC	NM_006251
	Reverse Sequence	CCGATCTCTGTGGAGTAGCAGT	
FOXP2	Foward Sequence	TGGATGACCGAAGCACTGCTCA	NM_148898
	Reverse Sequence	TGGGAGATGGTTTGGGCTCTGA	
WNT3	Foward Sequence	GCGTGTTAGTGTCCAGGGAGTT	NM_030753
	Reverse Sequence	TGAGGTGCATGTGGTCCAGGAT	
NPHP4	Foward Sequence	ATGGTGGTGAGTGGAGACATGC	NM_001291593
	Reverse Sequence	ATCTGGACGGTGGCAATGTGCT	
PLXNA1	Foward Sequence	ATGAGTGCCTCTTCCACATCCC	NM_032242
	Reverse Sequence	CAAAGTTGCCGTTCCACACGAC	
18S	Foward Sequence	GCAGAATCCACGCCAGTACAAG	NM_022551
	Reverse Sequence	GCTTGTTGTCCAGACCATTGGC	
GAPDH	Foward Sequence	GTCTCCTCTGACTTCAACAGCG	NM_001256799
	Reverse Sequence	ACCACCCTGTTGCTGTAGCCAA	
