# Supplementary material for: Validation of Gene Expression Patterns for Oral Feeding Readiness: Transcriptional Analysis of Set of Genes in Neonatal Salivary Samples
Source: Genes (Basel). 2024 Jul 18;15(7):936. doi: 10.3390/genes15070936 (PMC11275400; doi:10.3390/genes15070936)
Supplement: Supplementary file 1 [file genes-15-00936-s001.zip › File S2.pdf]

Additional File S2

RNA concentration data, 260/280 and 260/280absorbance ratios.

	[RNA] ng/uL		Ratio 260/280	Ratio 260/230
Patient	Nanodrop	Qubit		
1,1	289,66	133,83	1,95	1,88
1,2	262,56	125,45	1,99	1,78
1,3	288,46	109,81	2	1,88
1,4	282,46	107,73	2,1	1,78
1,5	249,66	96,38	2	1,88
2,1	312,66	118,18	2	1,68
2,2	307,76	106,49	2	2,58
2,3	298,36	123,23	1,97	1,77
2,4	277,96	106,17	1,99	1,72
2,5	310,06	107,28	1,98	1,73
3,1	327,33	115,26	2,01	1,69
3,2	294,41	101,87	2	1,68
3,3	210,75	92,92	2	1,68
3,4	191,22	96,16	2,03	1,71
3,5	255,86	98,53	2,01	1,77
4,1	251,73	97,1	1,99	1,69
4,2	295,37	102,2	1,98	1,72
4,3	200,3	89,3	1,99	1,73
4,4	252,45	97,35	1,99	1,76
4,5	305,85	105,83	1,97	1,75
5,1	208,69	92,21	1,99	1,77
5,2	226,4	88,33	1,92	1,58
5,3	267,92	102,7	1,94	1,67
5,4	289,21	100,07	1,98	1,64
5,5	231,36	90,05	1,97	1,66
6,1	285,04	108,62	1,97	1,65
6,2	283,5	98,09	1,99	1,69
6,3	279,08	106,56	1,96	1,7
6,4	310,17	117,32	1,95	1,73
6,5	213,9	94,01	1,97	1,75
7,1	326,7	113,04	1,99	1,77
7,2	227,11	98,58	1,99	1,88
7,3	207,26	91,71	1,98	1,88
7,4	240,09	93,07	1,99	1,68
7,5	261,83	101,59	1,97	2,18
8,1	289,88	110,3	1,99	1,77
8,2	248,18	95,87	1,98	1,69
8,3	270,36	103,55	2,01	1,72
8,4	223,13	97,2	2	1,74

8,5	274,68	105,04	1,99	1,71
9,1	238,04	92,36	1,99	1,73
9,2	198,87	98,81	1,94	1,74
9,3	308,04	126,5882353	1,99	1,76
9,4	289,15	100,05	1,98	1,72
9,5	272,95	104,44	1,99	1,72
10,1	222,53	98	1,98	1,69
10,2	285,33	126,41	2	1,69
10,3	290,33	127,76	1,97	1,7
10,4	267,03	102,39	1,97	1,71
10,5	253,33	97,65	1,95	1,73
11,1	223,62	97,37	1,96	1,74
11,2	287,35	105,42	1,99	1,7
11,3	282,85	107,87	2,02	1,7
11,4	278,65	106,41	2,08	1,76
11,5	272,95	104,44	2,01	1,69
12,1	286,57	109,15	2	1,68
12,2	257,93	109,24	2	1,68
12,3	215,93	94,71	2,01	1,74
12,4	273,53	104,64	1,99	1,87
12,5	274,43	94,95	1,98	1,79
13,1	269,13	93,124	1,98	1,83
13,2	203,03	90,25	1,99	1,79
13,3	301,13	173,4	1,99	1,78
13,4	293,13	122,19	1,99	1,78
13,5	362,73	125,51	1,99	1,78
14,1	286,13	109,06	1,98	1,78
14,2	330,93	114,5	1,95	1,78
14,3	368,01	137,71	1,97	1,78
14,4	314,35	108,77	1,99	1,71
14,5	194,82	97,41	1,99	1,73
15,1	359,46	124,38	1,99	1,74
15,2	255,33	98,34	2	1,7
15,3	298,97	103,44	1,98	1,72
15,4	203,9	96,55	1,99	1,77
15,5	256,05	98,59	1,98	1,76
16,1	289,45	107,07	1,98	1,69
16,2	212,29	93,45	1,94	2,18
16,3	230	99,58	2	1,68
16,4	271,52	93,95	2,12	1,8
16,5	292,81	101,31	2,09	1,77
17,1	234,96	101,3	2,11	1,79
17,2	308,64	106,79	2,11	1,79
17,3	297,1	133,94	2,01	1,69
17,4	282,68	132,41	2	1,68

17,5	243,77	104,34	2	1,68
18,1	217,5	95,25	1,99	1,71
18,2	320,3	110,83	1,99	1,77
18,3	250,71	96,75	1,98	1,69
18,4	310,86	107,56	1,99	1,72
18,5	243,69	94,32	2	1,73
19,1	256,43	98,73	2,01	1,76
19,2	203,48	99,4	1,99	1,75
19,3	251,78	97,12	1,99	1,77
19,4	273,96	94,79	2,01	1,7
19,5	226,73	98,45	2,01	1,69
20,1	278,28	96,29	2	1,73
20,2	241,64	93,61	2	1,74
20,3	302,47	104,66	1,99	1,79
20,4	205,04	98,94	1,97	1,65
20,5	263,5	101,176	2,01	1,69
21,1	241,5	93,56	2,01	1,87
21,2	242,13	93,78	1,99	1,76
21,3	276,49	105,67	1,99	1,79
21,4	260,62	100,17	2,01	1,9
21,5	295,62	107,68	2,01	1,68
22,1	198,46	108,67	2	1,8
22,2	257,46	99,08	2	1,87
22,3	235,84	101,6	1,99	1,79
22,4	210,91	102,97	1,94	1,79
22,5	196,54	92,58	2,01	1,84
23,1	346,45	153,80	1,88	1,89
23,2	339,35	144,42	1,92	1,79
23,3	265,25	118,78	1,93	1,89
23,4	259,25	116,71	2,03	1,79
23,5	226,45	105,36	1,93	1,89
24,1	289,45	127,16	1,93	1,69
24,2	284,55	125,46	1,93	1,86
24,3	275,15	122,21	1,9	1,78
24,4	254,75	115,15	1,92	1,73
24,5	286,85	126,26	1,91	1,74
25,1	304,12	132,23	1,94	1,7
25,2	331,2	155,44	1,93	1,69
25,3	347,54	161,10	1,93	1,69
25,4	358,01	154,34	1,96	1,72
25,5	232,65	107,50	1,94	1,78
26,1	228,52	106,07	1,92	1,7
26,2	272,16	121,17	1,91	1,73
26,3	277,09	122,88	1,92	1,74
26,4	229,24	106,32	1,92	1,77

26,5	282,64	124,80	1,9	1,76
27,1	355,48	160,38	1,92	1,78
27,2	203,19	97,31	1,85	1,59
27,3	244,71	111,67	1,87	1,68
27,4	266	119,04	1,91	1,65
27,5	208,15	99,02	1,9	1,67
28,1	341,83	159,12	1,9	1,66
28,2	340,29	151,67	1,92	1,7
28,3	335,87	150,14	1,89	1,71
28,4	286,96	126,29	1,88	1,74
28,5	194,69	92,98	1,9	1,76
29,1	303,49	132,01	1,92	1,78
29,2	323,9	139,08	1,92	1,89
29,3	334,05	159,89	1,91	1,89
29,4	216,88	102,04	1,92	1,69
29,5	238,62	109,57	1,9	2,19
30,1	346,67	153,88	1,92	1,78
30,2	324,97	139,45	1,91	1,7
30,3	327,15	147,12	1,94	1,73
30,4	299,92	165,38	1,93	1,75
30,5	251,47	114,01	1,92	1,72
31,1	314,83	135,94	1,92	1,74
31,2	275,66	122,38	1,87	1,75
31,3	284,83	125,56	1,92	1,77
31,4	265,94	119,02	1,91	1,73
31,5	249,74	113,42	1,92	1,73
32,1	299,32	130,57	1,91	1,7
32,2	342,12	145,38	1,93	1,7
32,3	317,12	136,73	1,9	1,71
32,4	243,82	111,37	1,9	1,72
32,5	230,12	106,63	1,88	1,74
33,1	200,41	96,35	1,89	1,75
33,2	264,14	118,40	1,92	1,71
33,3	259,64	116,84	1,95	1,71
33,4	255,44	115,39	2,01	1,77
33,5	249,74	113,42	1,94	1,7
34,1	263,36	118,13	1,93	1,69
34,2	234,72	108,22	1,93	1,69
34,3	192,72	93,69	1,94	1,75
34,4	250,32	113,62	1,92	1,88
34,5	251,22	113,93	1,91	1,8
35,1	245,92	112,09	1,91	1,84
35,2	209,82	96,14	1,92	1,8
35,3	277,92	123,17	1,92	1,79
35,4	329,92	141,16	1,92	1,79
35,5	339,52	144,48	1,92	1,79

36,1	262,92	117,98	1,91	1,79
36,2	307,72	133,48	1,88	1,79
36,3	274,8	122,09	1,9	1,79
36,4	291,14	127,74	1,92	1,72
36,5	271,61	120,98	1,92	1,74
37,1	236,25	108,75	1,92	1,75
37,2	232,12	107,32	1,93	1,71
37,3	275,76	122,42	1,91	1,73
37,4	220,69	103,36	1,92	1,78
37,5	232,84	107,57	1,91	1,77
38,1	208,24	99,06	1,91	1,7
38,2	199,08	92,43	1,87	1,87
38,3	206,79	98,55	1,93	1,69
38,4	248,31	112,92	1,85	1,81
38,5	269,6	120,29	1,89	1,78
39,1	211,75	100,27	1,99	1,8
39,2	245,43	111,92	1,99	1,8
39,3	283,89	125,23	1,94	1,7
39,4	278,28	123,29	1,93	1,69
39,5	209,56	99,51	1,93	1,69
40,1	204,29	97,69	1,92	1,72
40,2	207,09	98,66	1,92	1,78
40,3	227,5	105,72	1,91	1,7
40,4	237,65	109,23	1,92	1,73
40,5	220,48	103,29	1,93	1,74
41,1	242,22	110,81	1,94	1,77
41,2	270,27	120,52	1,92	1,76
41,3	228,57	106,09	1,92	1,78
41,4	250,75	113,76	1,94	1,71
41,5	203,52	97,42	1,94	1,7
42,1	255,07	115,26	1,93	1,74
42,2	218,43	102,58	1,93	1,75
42,3	279,26	123,63	1,92	1,8
42,4	281,83	124,52	1,9	1,66
42,5	240,29	110,15	1,94	1,7
43,1	238,29	109,45	1,94	1,88
43,2	218,92	102,75	1,92	1,77
43,3	253,28	114,64	1,92	1,8
43,4	237,41	109,15	1,94	1,89
43,5	272,41	121,26	1,94	1,69
44,1	275,25	122,24	1,93	1,81
44,2	252,25	114,28	1,93	1,88
44,3	252,63	114,42	1,92	1,8
	287,7	99,55	1,87	1,8
44,5	266,33	95,15	1,94	1,7
45,1	232,12	100,31	1,88	1,77

45,2	275,76	95,41	1,91	1,8
45,3	325,69	112,69	1,87	1,89
45,4	212,84	93,64	1,88	1,69
45,5	328,24	113,57	1,87	1,73
Media	265,2329778	111,8704392	1,954844444	1,758222222
Standard Deviation	40,91972184	16,86757408	0,047996445	0,095928007
