# Supplementary figures and images for: Validation of Gene Expression Patterns for Oral Feeding Readiness: Transcriptional Analysis of Set of Genes in Neonatal Salivary Samples
Source: Genes (Basel). 2024 Jul 18;15(7):936. doi: 10.3390/genes15070936 (PMC11275400; doi:10.3390/genes15070936)

## Additional File S4

Melt curves of the PCR amplicons used in the RT-qPCR experiments.

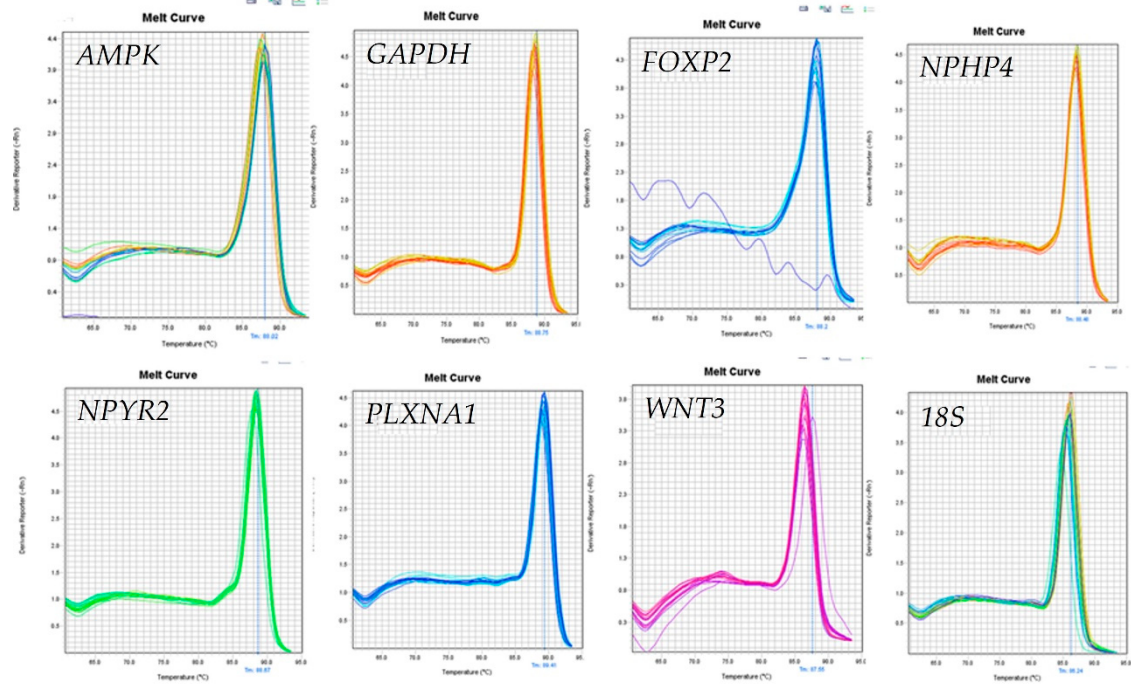

Supplement: Supplementary file 1 [file genes-15-00936-s001.zip › File S4.pdf]
